# 565. Pilot Study of Prolonged Post-Discontinuation Antibiotic Exposure (PDAE) to Ampicillin Among Low Birthweight Preterm Infants

**DOI:** 10.1093/ofid/ofac492.618

**Published:** 2022-12-15

**Authors:** Angelique E Boutzoukas, Angelique E Boutzoukas, Ryan Kilpatrick, Daniel Benjamin, Jennifer Le, Jennifer Le, Rachel Greenberg, Kelly Wade, Michael Cohen-Wolkowiez, Kanecia O Zimmerman

**Affiliations:** Duke University School of Medicine, Durham, North Carolina; Duke University School of Medicine, Durham, North Carolina; Duke University School of Medicine, Durham, North Carolina; Duke University Medical Center, Durham, North Carolina; University of California, San Diego Skaggs School of Pharmacy and Pharmaceutical Sciences, San Diego, California; University of California, San Diego Skaggs School of Pharmacy and Pharmaceutical Sciences, San Diego, California; Duke Clinical Research Institute, Durham, North Carolina; Children's Hospital of Philadelpia, Philadelphia, Pennsylvania; Duke University, Durham, North Carolina; Duke Clinical Research Institute, Durham, North Carolina

## Abstract

**Background:**

In the United States, 75% of preterm infants receive ampicillin after birth for evaluation of early onset sepsis. Excess antibiotic exposure in preterm infants is associated with morbidity and mortality. Pharmacokinetic (PK) simulations suggest that very low birthweight (< 1500g) infants receiving ampicillin may experience excess and prolonged therapeutic exposures to ampicillin after drug discontinuation, called post-discontinuation antibiotic exposures (PDAE). It is unknown if low birthweight ( > 1500g to < 2500g) infants that receive ampicillin experience prolonged PDAE.

**Methods:**

We conducted a pilot prospective PK clinical trial at a single tertiary care center. We enrolled infants with gestational age (GA) < 36 weeks and birthweight (BW) > 1500g and < 2500g who received ampicillin 200 mg/kg/day per standard of care. All infants received 6 ampicillin 66mg/kg doses administered Q8H within 24 hours following birth. Post-discontinuation PK samples were collected between 8-120 hours after the last dose of ampicillin. We performed descriptive statistics on patient characteristics, and graphically displayed ampicillin plasma concentrations following discontinuation. We defined therapeutic exposures as concentrations above the minimum inhibitory concentration (MIC) for Group B Streptococcus (0.25mcg/mL).

**Results:**

We analyzed 20 PK samples from 12 infants with median (IQR) GA of 32 weeks (31, 34) and BW of 1838g (1730, 2060). See Table 1 for clinical characteristics. All infants (N=8) with post-discontinuation samples collected within 48 hours of last dose had continued therapeutic ampicillin exposures, only 25% of infants had continued therapeutic exposures between 48 and 72 hours post last dose (N=4), and none had therapeutic ampicillin exposures beyond 72 hours post-dose (Figure 1). Samples (n=5) within 24 hours of last dose had median (IQR) ampicillin concentration of 13.2mcg/mL (9.2, 18.7); those within 25-48 hours (n=3) had median concentration of 0.9 mcg/mL (0.6, 2.2).

**TABLE 1.**
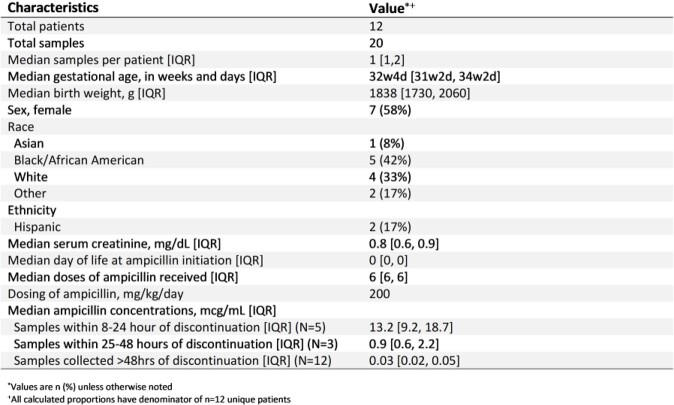
Enrolled Patient Characteristics and Plasma Ampicillin Concentrations

**Figure 1.**
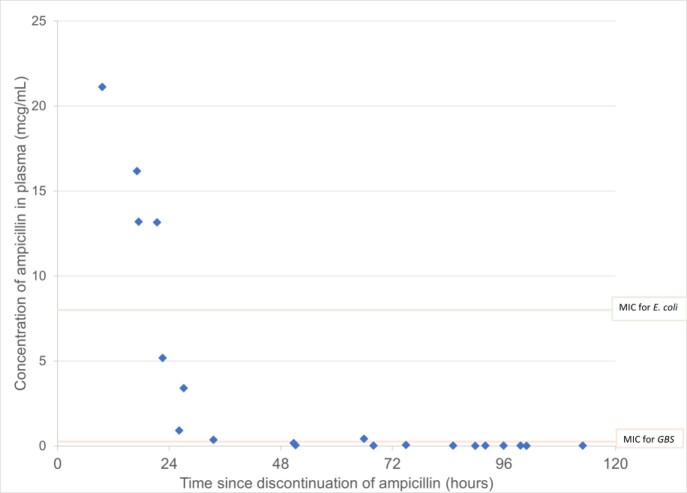
Ampicillin Post-Discontinuation Antibiotic Exposures in Low Birthweight Infants (1500g-2500g)

**Conclusion:**

In this small cohort of premature infants, exposures of ampicillin remained therapeutic for at least 24 hours post drug discontinuation and suggests that shorter duration of empirical ampicillin treatments may be warranted in this population.

**Disclosures:**

**Daniel Benjamin, Jr., MD PhD MPH**, Allergan: Advisor/Consultant|Melinta Therapuetics: Advisor/Consultant|Syneos Health: Advisor/Consultant **Rachel Greenberg, MD, MB, MHS**, Provepharm Inc: Advisor/Consultant|Tellus Therapeutics: Advisor/Consultant.

**Figure 1.** Ampicillin Post-Discontinuation Antibiotic Exposures in Low Birthweight Infants (1500g-2500g)

MIC = minimum inhibitory concentration. GBS = Group B Streptococcus. Each individual symbol represents one sample collected. MICs based on European Committee on Antimicrobial Susceptibility Testing (EUCAST) breakpoints (8 for E. coli, 0.25mcg/mL for Group B Streptococcus).

